# Volumetric Deficit Within the Fronto-Limbic-Striatal Circuit in First-Episode Drug Naïve Patients With Major Depression Disorder

**DOI:** 10.3389/fpsyt.2020.600583

**Published:** 2021-01-20

**Authors:** Yiran Zhang, Yun Yang, Licheng Zhu, Qing Zhu, Yuxi Jia, Lan Zhang, Qinmu Peng, Jiazheng Wang, Jia Liu, Wenliang Fan, Jing Wang

**Affiliations:** ^1^Department of Radiology, Union Hospital, Tongji Medical College, Huazhong University of Science and Technology, Wuhan, China; ^2^Hubei Province Key Laboratory of Molecular Imaging, Wuhan, China; ^3^Department of Neurology, Union Hospital, Tongji Medical College, Huazhong University of Science and Technology, Wuhan, China; ^4^School of Electronic Information and Communications, Huazhong University of Science and Technology, Wuhan, China; ^5^Clinical and Technical Solutions, Philips Healthcare, Beijing, China

**Keywords:** voxel-based morphometry, major depression, first episode, drug naïve, prefrontal cortex, limbic cortex, striatum

## Abstract

**Background:** Depression is a major psychiatric disorder and the leading cause of disability worldwide. Previous evidence suggested certain pattern of structural alterations were induced by major depression disorder (MDD) with heterogeneity due to patients' clinical characteristics and proposed that early impairment of fronto-limbic-striatal circuit was involved. Yet the hypothesis couldn't be replicated fully. Accordingly, this study aimed to validate this hypothesis in a new set of first-episode, drug naïve MDD patients and further explore the neuroimaging biomarker of illness severity using whole-brain voxel-based morphometry (VBM).

**Materials and Methods:** A total of 93 participants, 30 patients with first-episode medication-naïve MDD, and 63 healthy controls were enrolled in the study. VBM was applied to analyze differences in the gray matter volume (GMV) between these two groups. The correlation between the GMV of the identified brain regions and the severity of clinical symptoms quantified by the Hamilton Depression Scale (HAMD) was further conducted in the *post-hoc* analysis to confirm the role of GMV structural alteration in clinical symptoms.

**Results:** Our results revealed that the brain gray matter volume of the prefrontal lobe, limbic system, striatum, cerebellum, temporal lobe, and bilateral lingual gyri were significantly decreased in MDD patients compared with healthy controls. Besides, the HAMD scores were negatively correlated with GMV of the right insula and positively correlated with that of the right lingual gyrus.

**Conclusions:** Our findings provide robust evidence that gray matter structural abnormalities within the prefronto-limbic-striatal circuit are implicated in the pathophysiology of MDD at an early stage without confounding influence of medication status. Besides, our data suggest that the cerebellum, lingual gyrus, and fusiform gyrus should also be integrated into the brain alterations in MDD. Future synthesis of individual neuroimaging studies and more advanced statistical analysis comparing subfields of the aforementioned regions are warranted to further shed light on the neurobiology of the disease and assist in the diagnosis of this burdensome disorder.

## Introduction

Depression, which includes affective, cognitive, and somatic symptoms, is a major psychiatric disorder and a leading cause of disability worldwide. Depression dramatically affects the quality of life, and all age groups can suffer from major depressive disorder (MDD). In patients without chronic comorbidity, the average 1-year MDD prevalence is 3.2%, while in those with chronic physical conditions, the average 1-year prevalence ranges from 9.3 to 23.0% (1). Besides, the increased suicidal behaviors and secondary mental disorders are commonly present among patients with MDD (2). Consequently, large prevalence and high mortality lead to enormous social and economic burden (3). Considering this situation, it is urgent to accurately identify MDD and make a timely, accurate diagnosis so as to implement preventive measures ahead of time and provide beneficial mental health services.

Over the last few decades, increasing evidence from postmortem studies (4) and *in vivo* studies emerged that mood disorder can induce structural and morphometric changes of the brain, bringing about interests in the visualization of the disease at brain level. Voxel-based morphometry (VBM), now a widely used methods in neuroimaging studies, is particularly helpful in quantitatively identifying unexpected anatomic changes. Unlike regions of interests (ROIs), it assesses the entire brain, evading the need to make *priori* assumptions of the affected neural circuits (5). Previous VBM studies have reported a widespread of regions of abnormality (6). However, these results vary from study to study. Besides the demographical difference of ethnicity, age and sex and methodological variance such as field strength of MRI scanner across studies, another major heterogeneity lies in the fact that the number of episodes and treatment status differs among each study population (7). Both these two factors have been reported to confound the volumetric difference detected between depressive patients and healthy controls (7–10). Compared with healthy subjects, lower gray matter volume in the hippocampus was observed in recurrent patients but not first-episode patients (7), and Bora et al. demonstrated a significant reduction of GMV in the dorsomedial frontal cortex bilaterally in multiple-episode vs. first-episode patients (8). Studies on population with a higher fraction of anti-depressant treatment were shown to have a larger effect size of GMV reduction in the orbitofrontal cortex and subgenual prefrontal cortex (9). Others reported a tendency of normalization of GMV after anti-psychotic or anti-depressant medication, presented as an increase of GMV in the caudate (7), hippocampus (11), middle frontal gyrus and orbitofrontal cortex (10). To elucidate the core pathophysiology of the illness *per se*, it's thus paramount to control for these two factors and focus on patients at an early stage of the disease.

Recently, there has been emerging studies on the population of first-episode and medication-naïve MDD patients (10, 12–16). And subsequent meta-analyses (17–20) regarding this have been conducted. Yet no consistent findings can be reached. Some showed increased GMV in the prefrontal cortex (10, 13, 18), thalamus, striatum (14), insula (13, 17, 20) and limbic system (18, 20), while others (10, 15, 17, 19) reported decreased GMV within those regions and demonstrated additional areas affected, for example, postcentral gyrus (15) and cerebellum (16). Regardless of the direction of the volumetric alteration, certain affected regions are largely overlapped and several neural circuits involving these key nodes have been proposed to be impaired in the pathogenesis of MDD. Most pronounced of all is the fronto-limbic circuit and the fronto-subcortical circuit. The fronto-limbic circuit, primarily consisting of the orbitofrontal cortex, the anterior cingulate cortex, insula and limbic regions including the amygdala and hippocampus, is known as the brain's affective network, underlying the processing and regulation of emotions (21). The fronto-subcortical circuit, composed of the striatum, thalamus and prefrontal cortex, is implicated in emotional and cognitive processing, specifically reward-related cognition (21, 22). Indeed, this theory has been largely supported by convergent evidence from functional studies. Most replicated findings across functional studies suggest that MDD patients have resting state hyperactivity in the amygdala and the ventral components of the anterior cingulate cortex (19, 23). Aberrant functional connectivity was also observed within in both networks among depressive patients and was shown to be correlated with affect scores (24).

However, to our knowledge no original VBM study up to date corroborated the theory fully. In addition to the well-known heterogeneity of patients' clinical characteristics, this might also be possibly due to the paucity of sample size restricted by the difficulty in recruiting eligible patients (25) and selection bias of study population toward certain demographic features. And no consistent biomarker of symptom severity is identified across studies. Amygdala (20), hippocampus (12), insula (26), thalamus (14) and subgenual prefrontal cortex (9) were previously found to be correlated with severity in single study, respectively. Yet the results couldn't be replicated. Hence we performed this study in a new set of patients from the middle part of China, aiming to validate structural impairment in fronto-striatal-limbic circuit in MDD patients at an early stage independent from recurrent episodes and medication status and further explore the neuroimaging biomarker of illness severity, yielding more valid evidence to the current knowledge.

## Materials and Methods

### Participants

Thirty patients with MDD and sixty-three healthy control subjects were recruited at Wuhan Union Hospital between October 2018 and December 2019. All of them were right-handed. MDD patients were assessed through SCID-5 and diagnosed by an experienced specialist (QZ) in accordance with the Diagnostic and Statistical Manual of Mental Disorders-5 (DSM-5) criteria for MDD. Other inclusion criteria for patients were as follows: (1) experiencing the first-episode depression; (2) age between 18 and 65 years old; (3) had a 17-item Hamilton Depression Scale (HAMD) scored 17 or greater on the day of their scan; (4) had never received any anti-psychotic or anti-depressant medication. Patients were excluded if they (1) met DSM-5 criteria for such disorders as bipolar disorder, persistent depressive disorder, schizophreniform disorder, primary substance-induced psychotic disorder, any personality disorder and dementia; (2) had previous and current use of substance per the DSM-5; (3) had a history of serious head injury with a loss of consciousness, past or current neurological disease, or neurodegenerative disease; (4) had an MRI contraindication including pregnancy. Considering the high comorbidity rate of depression with anxiety disorder (27) and the paucity of our sample size, we didn't exclude patients comorbid with anxiety, Instead, the severity of anxiety symptoms was measured by the Hamilton Anxiety Rating Scale (HAMA). 17-item HAMD was used to assess the severity of depression.

Sixty-three healthy controls from the same sociodemographic environment as the patients were recruited by posters in the local communities. They all underwent SCID-5 to rule out current or past psychiatric or neurological diseases. The HAMD and Patient Health Questionnaire-9 (PHQ-9) were used to confirm their healthy status further. Other exclusion criteria were family history of psychiatric diseases among their first-degree relatives, serious medical diseases as well as MRI contraindications.

The Medical Ethics Committee approved the present study of Tongji Medical College of Huazhong University of Science and Technology (No. 20200301-01). Written consent was obtained from all participants after a comprehensive description of the study.

### MRI Data Acquisition

The imaging data were performed on a 3T MRI system (Ingenia 3.0T CX, Philips Healthcare, Best, Netherlands) equipped with a 32-channel head coil. Participants were asked to lay still with ears covered by foam-cushioned headphones in an attempt to both curtail the noise and immobilize the head. For each participant, a high-resolution T1 structural MRI imaging was acquired using a 3D fast gradient echo sequence (“Turbo Field Echo,” TR = 11.2 s, TE = 5.1 s, FA = 8°, acquired over a field of view of 384^*^384, slice thickness 0.7 mm, slices number = 258). All MRI images were visually inspected for artifacts and gross abnormalities by an experienced neuroradiologist.

### Data Processing and Analysis

Data processing was performed using the CAT12 toolbox (http://www.neuro.uni-jena.de/cat/) within the SPM12 software package (SPM12, http://www.fil.ion.ucl.ac.uk/spm) running under MATLAB (Math Works, Natick, MA, U.S.A.). The corrected images in native spaces were first spatially normalized and then segmented into gray matter, white matter, and cerebrospinal fluid within the same generative model. Next, the segmented images were iteratively registered by the Diffeomorphic Anatomical Registration Through Exponentiated Lie algebra toolbox (DARTEL) to improve the intersubject registration (28). Meanwhile, a template specific for the group of individuals was created in this step. After initial affine registration of the GM DARTEL templates to the tissue probability maps in Montreal Neurological Institute (MNI) space, non-linear warping of GM images was performed to the template in MNI space and then employed in the modulation step to ensure that relative volumes of GM were preserved following the spatial normalization procedure. Finally, the resulting gray matter images were smoothed with an isotropic Gaussian kernel of 10-mm full width at half maximum (FWHM) to exploit the partial volume effects and increase the signal-to-noise ratio.

### Statistical Analysis

Mann-Whitney U test and Chi-square tests were used to compare demographic and clinical severity (HAMD) between MDD patients and HC with SPSS 23.0 software (IBM Corp, Armonk, NY). Voxel-wise comparison of GMV between the two groups was performed through two-sample *t* test implemented in the Data Processing and Analysis for Brain Imaging (DPABI) software (http://rfmri.org/dpabi), after regressing out age, gender and total brain volume as covariates. AlphaSim procedure was implemented to control for multiple statistical testing within the entire brain (29). A cluster-level false-positive detection rate at *p* < 0.05 using a voxel-level threshold of *p* < 0.01 was maintained. The cluster extend (k), which was set as 5171 voxels, was empirically determined by Monte Carlo simulations (*n* = 1,000 iterations). Identified cluster regions were anatomically labeled with reference to the AAL-definitions (30).

To assess the correlation of the regional gray-matter volume identified above with the severity of clinical symptoms measured by HAMD, we also performed a multiple regression analysis. In this *post-hoc* analysis, global brain volume, age, and gender were included as covariates. Exploration of the correlation with anxiety symptoms was conducted in the same manner. AlphaSim procedure was carried out to control for multiple statistical testing as previously described.

## Results

To investigate the brain structural alterations in patients with MDD, we conducted this cross-sectional study. We then analyzed demographic and MRI imaging data as well as the correlation with clinical severity (HAMD), using SPSS 23.0, SPM12 software, and AlphaSim procedure. Our study showed that the GMV was reduced in several brain regions. Besides, clinical severity was negatively related to the right insula volume and positively related to lingual gyrus volume.

### Demographic and Clinical Characteristics

Thirty patients with MDD and 63 healthy controls were included in this study according to the inclusion criteria. The demographic and clinical characteristics of the sample are provided in detail in Table 1. There was no significant difference in age (*p* = 0.43) and sex (*p* = 0.45) between the patients' group and the healthy controls' group. As expected, the HAMD score in patients' group was significantly higher compared to the healthy controls' group (*p* < 0.001).

**Table 1:** Demographic and clinical characteristic of all the participants.

	**Healthy controls (*n* = 63)**	**Patients (*n* = 30)**	**Statistical significance**
Age (years)	23.0 (22.0–29.0)	25.0 (21.8–48.3)	0.43^a^
Sex (M/F)	24/39	9/21	0.45^b^
Years of education^c^	16.0 (16.0–17.0)	12.5 (9.3–15.8)	<0.001^a^
HAMD score	4.0 (2.0–6.0)	26.5 (22.8–32.0)	<0.001^a^
HAMA score^d^	3.0 (1.0–6.0)	28.0 (25.0–33.0)	<0.001^a^

a *The variables were described by median and quantile and were tested by Mann-Whitney U test*.

b *The variables were described by the numbers and were tested by χ^2^ test*.

c *Data was missing for 6 patients in MDD patients group*.

d *Scores were not available for 1 patients in MDD patients group*.

*HAMD, Hamilton Depression Rating Scale; HAMA, Hamilton Anxiety Rating Scale*.

### Imaging Analysis of Gray Matter Volume—VBM

**Figure 1:**
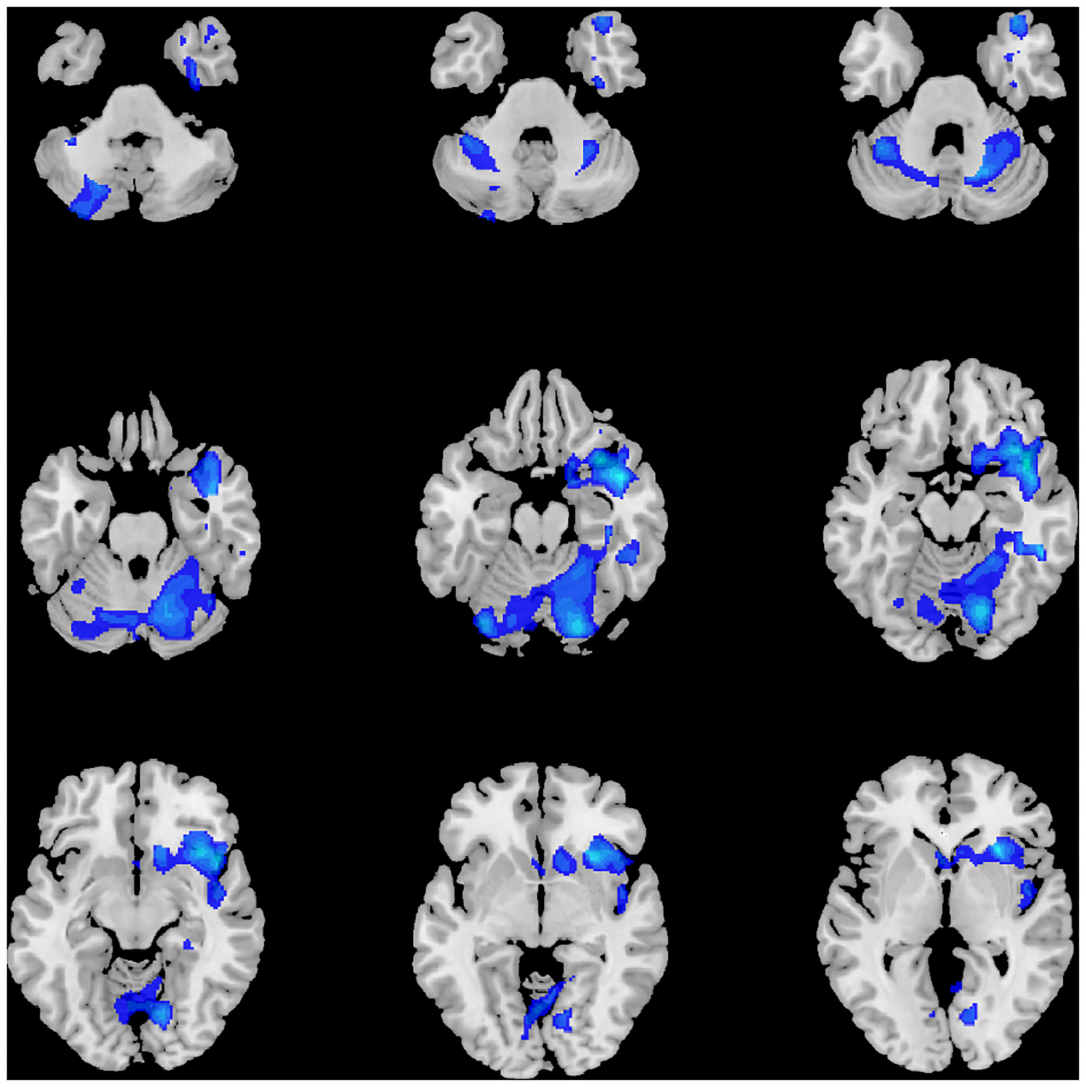
Significant volume differences between MDD and HC.

As displayed in Table 2 and Figure 1, at the threshold of individual voxel *p* < 0.01 (AlphaSim corrected) and extend cluster size > 5171 voxels, the difference in image data was significant between MDD patients' and healthy controls' group. Notably, the GMV change was identified in the following two clusters of the brain region.

**Table 2:** Clusters of gray matter volumes were significantly reduced in all patients compared to healthy controls.

**Anatomical location**	**Cluster size^a^ (voxels)**	**Peak MNI coordinates**			***P*^b^**
		**x**	**y**	**z**	
Limbic system	6,583	42	7.5	−16.5	<0.01
Pre-frontal lobe					
Basal ganglia					
Right superior temporal gyrus^c^					
Cerebellum	12,489	19.5	−78	−18	<0.01
Right ParaHippocampal gyrus					
Temporal lobe					
Bilateral lingual gyri^d^					

a *The number of voxels in every cluster*.

b *AlphaSim corrected*.

c *Limbic system here involves right insula, right superior and middle temporal poles (peak voxels), and right olfactory gyrus; pre-frontal lobe involves right rectus and right inferior orbitofrontal gyrus; basal ganglia involves right putamen and caudate nuclei*.

d *Cerebellum here refers to right lobule IV, V, VI (peak voxels) and crus 1, left lobule VI, VIIb, VIII, crus I and crus II, and lobule IV, V, VI, and VII of the vermis (AAL); temporal lobe refers to right inferior temporal gyrus and bilateral fusiform gyri*.

The first cluster of brain region with reduced GMV (6583 voxels, Peak MNI coordinates: x = 42, y = 7.5, z = −16.5) was located in the right brain regions and centered in the insula, extending antero-inferiorly to the right superior and middle temporal poles, olfactory gyrus, gyrus rectus, and inferior orbitofrontal gyrus, medially to the right putamen and caudate nuclei, as well as laterally to the right superior temporal gyrus.

The second cluster of brain region detected with reduced GMV (12,489 voxels, Peak MNI coordinates: x = 19.5, y = −78, z = −18) was bilateral and centered in the cerebellum, involving right lobule IV, V, VI, and crus 1, left lobule VI, VIIb, VIII, crus I and crus II, and lobule IV, V, VI, and VII of the vermis according to Anatomical Automatic Labeling (AAL), spanning antero-superiorly from bilateral lingual gyri to the right parahippocampal gyrus, and then laterally to bilateral fusiform gyri and the right inferior temporal gyrus.

### The Correlation of the GMV and HAMD Scores

**Figure 2:**
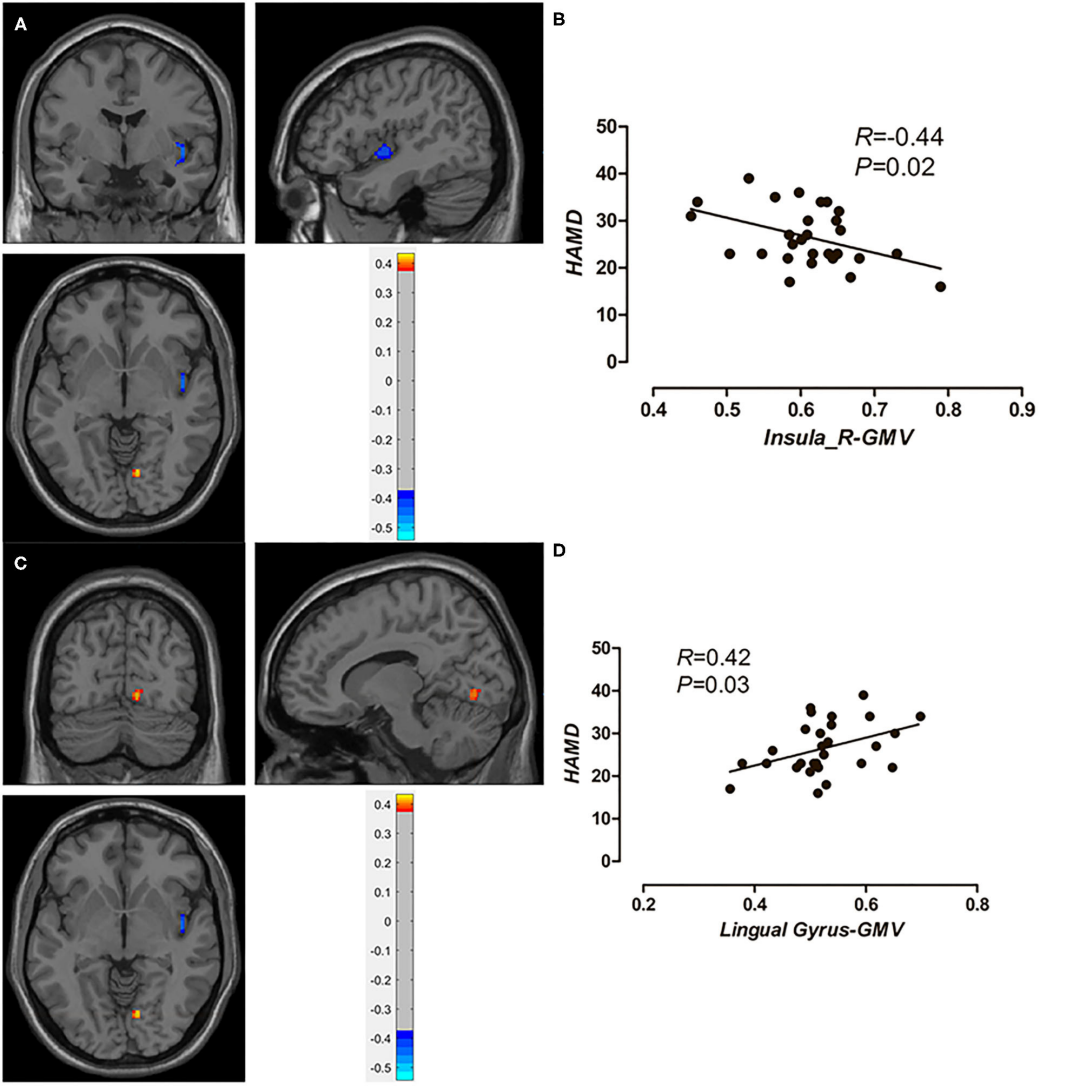
The negative correlation between GMV of the right insula and HAMD scores in MDD patients **(A,B)**; a positive correlation between GMV of the right lingual gyrus and HAMD scores in MDD patients **(C,D)**.

We defined the brain regions of these two clusters as regions of interest and conducted the *post-hoc* analysis. As Figure 2 shows, after controlling for age and gender, the GMV in the right insula was negatively correlated with the HAMD scores, and the GMV in the right lingual gyrus was positively correlated with the HAMD scores. A positive correlation (r = 0.58, *p* < 0.01) was observed between the GMV of the right cerebellum and HAMA scores (figure not presented).

## Discussion

The present study examined the structural alterations in a new set of first episode, medication-naïve MDD patients from the middle part of China. Highlighting a decrease of GMV in the right prefrontal cortex (including the orbitofrontal cortex, gyrus rectus), striatum, limbic lobe (i.e., insula, olfactory gyrus, parahippocampus, and temporal pole), bilateral lingual gyrus, fusiform gyrus as well as the cerebellum, our results validated the hypothesis that the structural alteration within the fronto-limbic-striatal circuit can be detected at an early stage independent from treatment status and recurrent episodes, confirming a profound role this network has in the pathophysiology of MDD. Besides, we detected a negative and positive association of clinical severity (HAMD) with the right insula volume and bilateral lingual gyrus volume, respectively. These results add valid evidence to our current knowledge of the altered brain structure of MDD and can further be synthesized to provide validated neuro-biomarkers to assist in the diagnosis of this burdensome disease.

### Prefronto-Limbic-Striatal Circuit

In the present study, we found that MDD patients had a lower GMV in the right inferior orbitofrontal gyrus and gyrus rectus, corresponding to the orbitofrontal cortex (OFC), which is a major component of prefrontal cortex (PFC). OFC is characterized by connections with several sensory areas, responding to multimodal sensory stimuli in relation to their rewarding or aversive qualities (31), thus having an indispensable role in the emotion and reward circuit (32). Damage to the OFC impairs emotion-related learning, emotional behavior, and subjective affective state. Indeed, prior histopathologic study (33) and neuroimaging studies (34) have reported on gray matter reduction in depressed patients, which was consistent with our results. Besides, decreased cerebral blood flow (35) and aberrant functional connectivity (36) have been reported in MDD patients in this specific region. Notably, the right side (32, 34, 36) seems to be prominently affected, which might be explained by the fact that the right OFC is more activated by punishment than positive rewards, resulting in increased responses to negative stimuli (right hemisphere) and decreased responses to positive stimuli (left) that are typically found in depressed patients (32).

GMV reductions of the caudate and putamen in depressed patients have also been identified in the study. Although the structural change in the striatum is not accordant across studies with some detecting diminished GMV (14, 27, 37, 38), some revealing increased GMV (19) and some failing to detect such GM abnormality, possible explanations could be the heterogeneity of demographic features of studied subjects, namely the medication status, illness severity and chronicity (14, 38). Our results revealed a diminished volume in the striatum in the first episode, untreated MDD, which was consistent with findings reported by Lu et al. (14). Furthermore, a genome-wide association study that identified 11 loci linked with anhedonia, which predicted a smaller volume in striatum, demonstrated a genomic basis for striatal involvement in depression (39).

Decreased GMV in the right insula, temporal pole, olfactory gyrus, and parahippocampus were also detected in our study. These areas are essential components of the limbic system implicated in the regulation of memory, emotion, and behavior, such as integration of visceral sensation and emotion, autobiographical memories, and introspective self-directed thinking (40). Previously, both anatomic and functional changes have been detected within the limbic system in patients with MDD. VBM studies showed altered GMV (7, 19) in the limbic system. Functionally, aberrant brain regional homogeneity (41) and functional connectivity have been reported. For example, He et al. found abnormal functional connectivity between the left and right amygdala/hippocampus, which were correlated with sadness (42). Besides, a translational study using MRI in animals found the underlying changes of serotonergic neuroplasticity in these areas, providing neurobiological evidence for structural and functional alterations in depressive patients (43).

Insula has been suggested as a trait-related biomarker (17), as lower GMV in this region was identified in various MDD samples, including FE patients (29, 34), medicated patients (44), current, and even remitted patients (45). The human insula is implicated in a wide range of cognitive, emotion, and somatosensory activity. An fMRI study detected increased insular activation to facial expressions of disgust in MDD (46), reflecting an emotion processing bias. Local connectivity reduction (47) and GMV reduction (48) in the insula was positively correlated with the magnitude of anxiety in MDD. Besides, depressed patients with increased appetite had a lower anterior insular surface area, substantiating insula's role in interoceptive awareness as well as reward processing and emotion regulation (49). However, as opposed to being a trait-dependent biomarker of MDD, the whole insula volume was shown to be reduced across other psychiatric disorders in one recent meta-analysis (5), questioning its diagnostic specificity. Thence, researchers indicate it might be certain subfield of insula such as the anterior portion that is MDD-related (26). This hypothesis also aids in explaining another paradoxical finding. While together with one previous research (46), our results demonstrated a negative correlation between insula volume and the severity of the illness, one study observed a gradient of negative to positive weights from the anterior to posterior sections of insula using machine learning methods (26). Future evaluation of GMV dissecting insula into subfields will help to elucidate this uncertainty.

Accordingly, the impairment of the fronto-limbic-striatal circuit can be detected in MDD patients as early as during the first episode and without the influence of medication. As suggested by prior studies, this neural system is strongly linked with clinical symptoms, specifically with the fronto-striatum system implicated in the reward-related impairment and the fronto-limbic circuit underlying dysphoria and disrupted cognitive control (21). The fronto-striatum system, also known as the reward/motivation circuit, was reported to be altered both anatomically and functionally (21, 37, 50, 51), which in turn is associated with core symptoms such as social anhedonia (21), cognitive impairment (51), and psychomotor retardation (50). Strikingly, deep brain stimulation therapy targeting the striatum showed improvement of cognitive performance in MDD subjects', which was, in part, driven by PFC as indicated by increases in theta (5–8 Hz) oscillations in both medial and lateral PFC (52). The limbic system's connections with the frontal cortex have been extensively studied. Given the number of studies revealing functional disruption inside the system, researchers have revised three related subnetworks as the neural substrates for depression-the ventral limbic affective network dedicated to excessive negative mood, the default mode network for depressive rumination and the dorsal cognitive control network for cognitive deficits (21, 40). Of note, with antidepressant treatment, the aforementioned aberrant brain connectivity can be reported, which corroborates the fronto-limbic circuit's crucial role in MDD (21). Moreover, a growing literature involving both VBM (37) and DTI (53) investigations has revealed the fronto-limbic-striatal circuit's critical association with suicidal behavior. Therefore, our study provides structural evidence and underpins the importance of the abnormal fronto-limbic-striatal network in the pathogenesis of MDD.

### Lingual and Fusiform Gyrus

Lingual gyrus, which continues on to the fusiform gyrus, joins the parahippocampal gyrus and is thought to form emotion-limbic circuits with the adjacent limbic system, linking to the recollection of visual memory and emotional processing (54). In the context of MDD, where subjects have impaired social functioning, notably emotional facial processing, GM structural abnormalities in the lingual and fusiform cortices were found (27, 55). These findings are consistent with our data showing that patients with first-episode medication-free MDD had less GMV in bilateral lingual and fusiform gyri. Furthermore, functional abnormalities, including the altered amplitude of low-frequency fluctuation (56) and weakened connectivity (32), were reported within these two regions in depressed subjects. In line with these findings, disturbed connectivity between prefrontal and visual association areas, including the fusiform gyrus, was found to be associated with working memory deficit (57) identified in patients with MDD. Taken together, the previous and present findings support that impaired lingual and fusiform gyri under the control of the prefrontal network underlie the pathogenesis of MDD and may present as a deficit in visual memory, working memory and emotional bias.

Opposite to the previous study, which suggested larger lingual volume as an indicator for preserved cognitive function for antidepressant responses (44), our findings revealed a positive correlation between the GMV of the right lingual gyrus and HAMD score. Accordingly, we hypothesized that the recuperation of cognitive function by antidepressant is not a reversion to the normal structure pathologically; yet, further studies are needed to confirm this hypothesis.

### Cerebellum

Our results revealed that MDD patients have a smaller GMV in the bilateral cerebellum, which is in accordance with several recent VBM studies (34, 58) as well as a computational meta-analysis (6). The cerebellar function has traditionally focused on the motor domain, while an upsurge of multidisciplinary evidence has shown its role in higher-order cognitive and affective function (59, 60), particularly the posterior lobe. The present findings warrant further investigation to clarify its significance.

The present study has several limitations. Our sample size was small, which may limit the generalizability of our analysis. However, recruiting eligible patients with mood disorders through clinical routes have been shown to be challenging, especially in the subgroup of untreated population where individuals are likely to lose contact with health care services (25). Future studies recruiting subjects from online public advertisements may benefit from larger sample size (25). Besides, MDD subjects included in the study were all of Asian ethnicity, specifically the Han people, so the results should be extrapolated with caution to other ethnic groups. Furthermore, we didn't exclude MDD patients comorbid with anxiety disorder from our study due to our limited sample size. Indeed, a significant difference of the severity of anxiety symptoms assessed by Hamilton Anxiety Scale was observed between the patient group and the control group, confirming the high comorbidity rate with anxiety among depressive patients (27) and there was a positive correlation between anxiety severity and the GMV of the right cerebellum, which might have confounded with our findings in the cerebellum. To elucidate MDD's unique pathogenesis, further studies may consider acquiring patients' anxiety status and clarifying the anxiety type. Lastly, restricted by the current method applied in this study, we weren't able to make comparisons of voxels in subfields of regions and study the more complex pattern of volumetric alteration. Nevertheless, we were able to replicate the findings found in those studies using machine learning techniques (6, 26). Future investigation with more advanced statistical methods are warranted to dig deeper about the subtle structural cerebral changes in depressive patients.

## Conclusion

Our study revealed that structural abnormalities in the prefrontal cortex, limbic lobe and striatum may be presented at an early stage of MDD without confounding influence of medication status, thus providing robust evidence of the prefronto-limbic-striatal circuit's mediation in MDD. Besides, our data suggest that the cerebellum, lingual gyrus, and fusiform gyrus may also contribute to the pathophysiology of MDD. Future synthesis of individual neuroimaging studies and more advanced statistical analysis comparing subfields of the aforementioned regions are warranted to further shed light on the neurobiology of the disease and assist in the diagnosis of this burdensome disorder.

## Data Availability Statement

The raw data supporting the conclusions of this article will be made available by the authors, without undue reservation.

## Ethics Statement

The studies involving human participants were reviewed and approved by the Medical Ethics Committee of Tongji Medical College of Huazhong University of Science and Technology. The patients/participants provided their written informed consent to participate in this study.

## Author Contributions

JL, WF, and JingW conceived of the study. JL, JingW, QZ, YZ, YY, LZhu, YJ, and LZha contributed to recruitment. WF, QP, and JiazW contributed to data analysis. YZ and YY wrote the first draft of the manuscript. JL and JingW contributed to manuscript revision and approval. All authors contributed to the article and approved the submitted version.

## Conflict of Interest

The authors declare that the research was conducted in the absence of any commercial or financial relationships that could be construed as a potential conflict of interest.

## Abbreviations

GM: Gray Matter
GMV: Gray Matter Volume
HAMD: Hamilton depression scale
MDD: Major Depression Disorder
OFC: Orbitofrontal Cortex
PFC: Prefrontal Cortex
ROIs: Regions of Interests
VBM: Voxel-Based Morphometry.
